# Bacterial Pathogens and Antibiotic Resistance in Bloodstream Infections in Tunisia: A 13-Year Trend Analysis

**DOI:** 10.3390/tropicalmed10060164

**Published:** 2025-06-12

**Authors:** Lamia Kanzari, Sana Ferjani, Khaoula Meftah, Mariem Zribi, Sonda Mezghani, Asma Ferjani, Yosra Chebbi, Manel Hamdoun, Hajer Rhim, Yosr Kadri, Siwar Frigui, Emna Mhiri, Asma Ghariani, Nour Ben Ayed, Faouzia Mahjoubi, Yomna Ben Lamine, Salma Kaoual, Basma Mnif, Habiba Naija, Manel Marzouk, Sarra Dhraief, Hela Karray, Jaya Prasad Tripathy, Bobson Derrick Fofanah, Safa Bouwazra, Hajer Battikh, Ramzi Ouhichi, Lamia Thabet, Jalel Boukadida, Farouk Barguellil, Sophia Besbes, Leila Slim, Maha Mastouri, Olfa Bahri, Wafa Achour, Adnene Hammami, Hanen Smaoui, Ilhem Boutiba-Ben Boubaker

**Affiliations:** 1 National Reference Laboratory on Antimicrobial Resistance Surveillance, Laboratory of Microbiology, Charles Nicolle Hospital, Tunis 1006, Tunisia; ferjsana@yahoo.fr (S.F.); aferjani76@gmail.com (A.F.); ilhem.boutiba@gmail.com (I.B.-B.B.); 2 Research Laboratory “Antimicrobial Resistance” LR99ES09, Faculty of Medicine of Tunis, University of Tunis El Manar, Tunis 1007, Tunisia; 3 Laboratory of Microbiology, Bechir Hamza Children’s Hospital, Tunis 1006, Tunisia; meftahkhaoula@gmail.com (K.M.); hanen.smaoui@gmail.com (H.S.); 4 Laboratory of Microbiology, La Rabta Hospital, Tunis 1007, Tunisia; meriamzribimiled@gmail.com; 5 Laboratory of Microbiology of Sfax University Hospital Center, Sfax 3000, Tunisia; maalejsenda69@gmail.com (S.M.); bayednour@yahoo.fr (N.B.A.); mahjoubi_faouzia@medecinesfax.org (F.M.); basmamnif@gmail.com (B.M.); hela_kakim@yahoo.fr (H.K.); hammami.adnene@gmail.com (A.H.); 6 Research Laboratory LR03SP03 “Micro-Organisme et Pathologie Humaine”, Faculty of Medicine, University of Sfax, Sfax 3029, Tunisia; 7 Laboratory Department, Bone and Marrow Transplantation Center, Tunis 1006, Tunisia; yos.chebbi@gmail.com (Y.C.); siwar.frigui92@gmail.com (S.F.); safa.bouwazra@hotmail.com (S.B.); wafaachour@gmail.com (W.A.); 8 Laboratory of Clinical Biology, Aziza Othmana Hospital, Tunis 1008, Tunisia; hamdoun.zahmoul@gmail.com (M.H.); olfa.bahri@fmt.utm.tn (O.B.); 9 Laboratory of Microbiology, Fatouma Bourguiba Hospital, Monastir 5000, Tunisia; hajer.rhim@gmail.com (H.R.); yosrguedri@gmail.com (Y.K.); mastourimaha@yahoo.fr (M.M.); 10 Laboratory of Transmissible Diseases and Biologically Active Substances LR99ES27, Faculty of Pharmacy, University of Monastir, Monastir 5000, Tunisia; 11 Laboratory of Microbiology, Abderrahmen Mami Hospital, Ariana 2080, Tunisia; emna.mehiri@tunet.tn (E.M.); ghariani_as@yahoo.fr (A.G.); leilaslimsaidi@gmail.com (L.S.); 12 Laboratory of Microbiology, Mohamed Kassab Orthopedics Institute, La Manouba 2010, Tunisia; yomnabenlamine@gmail.com (Y.B.L.); salmakaoual88@gmail.com (S.K.); sophia.besbes@yahoo.fr (S.B.); 13 Laboratory of Microbiology, Military Hospital, Tunis 1008, Tunisia; naijahabiba@gmail.com (H.N.); farouk.barguellil@gmail.com (F.B.); 14 Laboratory of Microbiology, Farhat Hached Hospital, Sousse 4000, Tunisia; mnmarzouk@gmail.com (M.M.); jjboukadida@gmail.com (J.B.); 15 Laboratory of Clinical Biology, Burn and Trauma Center, Ben Arous 2013, Tunisia; dhraiefsarra@gmail.com (S.D.); thabetlamia@gmail.com (L.T.); 16 All India Institute of Medical Sciences, Nagpur 441108, India; jtripathy@aiimsnagpur.edu.in; 17 World Health Organization Country Office, Freetown P.O. Box 529, Sierra Leone; fofanahb@who.int; 18 Unit of Laboratories, Ministry of Health, Tunis 1006, Tunisia; hajerbattikh@yahoo.fr; 19 Faculty of Pharmacy, University of Monastir, Monastir 5000, Tunisia; 20 World Health Organization Country Office, Tunis 1082, Tunisia; ouhichir@who.int

**Keywords:** operational research, antimicrobial resistance surveillance, carbapenem resistant *Enterobacterales*, SORT-IT, GLASS

## Abstract

The antimicrobial resistance (AMR) surveillance network has been monitoring bloodstream bacterial pathogens and their resistance since 1999 in Tunisia. We report the long-term trends in the distribution of bloodstream bacterial pathogens and their resistance patterns from this surveillance database. We analyzed antibiotic resistance rates in 11 tertiary teaching hospital laboratories under the AMR surveillance network during 2011–2023, focusing on six priority bacterial pathogens, using the Cochrane–Armitage test for trend analysis. Of 22,795 isolates, *K. pneumoniae* (38.5%) was the most common, followed by *S. aureus* (20.4%), *E. coli* (13.6%), and *A. baumannii* (10.3%). Carbapenem resistance was highest in *A. baumannii* (77%), followed by *Pseudomonas aeruginosa* (29.3%), *K. pneumoniae* (19.4%), and *Enterobacter cloacae* (6.8%). Carbapenem-resistant *Enterobacterales* and third-generation cephalosporin-resistant *Enterobacterales* (3GCREB) increased from 10.6% to 26.3% (*p*-value < 0.001), and from 39% to 50.2%, respectively, during 2011–2023 (*p*-value < 0.001). Vancomycin resistance (38.3%) and the emergence of linezolid resistance in 2019 (2.4%) were reported in *E. faecium* isolates. Resistance to carbapenems and 3GC is a major challenge to controlling BSI in Tunisia. The national AMR surveillance network helps monitor annual patterns and guides empirical therapy. An integrated database combining clinical profiles and resistance data via real-time data-sharing platforms could improve clinical decision-making.

## 1. Introduction

Bloodstream infections (BSIs) are serious life-threatening infections that significantly contribute to morbidity, mortality, and healthcare costs worldwide [1]. Bacterial pathogens account for over 90% of all bloodstream infections (BSIs), with a considerable proportion arising from the six priority pathogens identified by the World Health Organization (WHO) [2,3]. Unfortunately, the global spread of antimicrobial resistance (AMR) and the emergence and spread of multidrug-resistant (MDR) bacterial strains, both in hospital and community settings, further complicate the management of these infections [4]. In 2021, bacterial AMR alone killed 1.14 million people (95% uncertainty interval 1.00–1.28), with nearly three-quarters of these deaths being caused by six common bacterial pathogens, also known as the ESKAPE pathogens [5,6].

In the Eastern Mediterranean Region, antimicrobial-resistant isolates are being increasingly reported, with carbapenem-resistant *Acinetobacter* species being the highest (70.3%), followed by *K. pneumoniae* resistant to third-generation cephalosporins (66.3%) in 2019 [7]. BSIs remain a major health burden in the region, with high rates of morbidity, mortality, and prolonged hospital stay, especially among vulnerable populations [8]. BSIs claimed nearly 159,000 lives and loss of 6.7 million Disability-Adjusted Life Years (DALYs) in the Middle East and North Africa (MENA) Region in 2019 [9].

Tunisia, located in the MENA region, has one of the highest rates of antibiotic consumption, with 38 defined doses per 1000 individuals per day. This rate is second only to Greece within the region and is approximately four times higher than the global average [10]. This, coupled with factors such as limited infection prevention and control measures, sanitation, and hygiene, could further accelerate the high AMR rates in the country. According to the latest report of the Global Antimicrobial Resistance and Use Surveillance System (GLASS) in 2020 in Tunisia, a substantial proportion of *K. pneumoniae* isolates from blood cultures were resistant to cefotaxime (68.4%) and carbapenems (27.7%). *A. baumannii* strains isolated from blood were resistant to carbapenems in 82.5% of cases, whereas *S. aureus* strains resistant to methicillin ranged from 10 to 30% [11]. The proportion of resistant isolates exhibiting these specific AMR phenotypes is concerning and has critical implications for clinical management and patient survival, necessitating immediate action.

In view of this imminent threat, surveillance of bacterial pathogens causing BSI and their AMR patterns started in Tunisia in 1999 by a voluntary initiative from a group of university microbiologists who formed a surveillance network called “L’Antibiorésistance en Tunisie” (LART). It began with four laboratories from Tunisian university hospitals, and since 2011, 11 laboratories have consistently tested and reported blood cultures from patients. These tests cover a wide range of bacterial pathogens and assess their sensitivity to various antibiotics following standard recommendations [12]. The test results were maintained using a laboratory information system (LIS). However, there is no published literature analyzing the laboratory surveillance data to document the long-term trends of the resistance patterns in Tunisia.

The previous literature in Tunisia on bacterial pathogens causing BSIs and their AMR patterns is limited to single-hospital studies over shorter periods and with limited sample sizes [13,14]. Information about long-term trends in AMR patterns of the most frequent bacteria causing BSI in Tunisia is useful for clinicians and policymakers in implementing empirical antibiotic protocols and infection control programs and informing them about emerging bacterial resistance threats.

Thus, we analyzed the laboratory-based AMR sentinel surveillance database in Tunisia to describe the long-term longitudinal trends in the (i) number of positive bacterial isolates, disaggregated by Gram-positive and Gram-negative pathogens; (ii) distribution of pathogens causing BSI; and (iii) their antimicrobial resistance patterns (among ESKAPE pathogens only) from to 2011 to 2023, with a focus on the specific AMR phenotypes of concern [third-generation cephalosporin-resistant (3GCREB) and carbapenem-resistant *Enterobacterales* CRE), carbapenem-resistant *A. baumannii* (CRAB), carbapenem-resistant *P. aeruginosa*, methicillin-resistant *S. aureus* (MRSA), and vancomycin-resistant *Enterococci* (VRE)], all of which are in line with the WHO global research priorities for AMR [15].

## 2. Materials and Methods

### 2.1. Study Design

This is a cross-sectional study involving the analysis of secondary aggregate data from the laboratory-based AMR surveillance network database.

### 2.2. Study Population

All blood culture bacterial strains were isolated and tested in the laboratories of Tunisian university hospitals that were part of the AMR surveillance network during the period 2011–2023.

### 2.3. Study Setting

- General setting

Tunisia is a North African country on the southern coast of the Mediterranean, inhabited by approximately 11 million people [14]. Tunisia has a great north–south environmental difference, defined by sharply decreasing southward rainfall from any point. Tunisia’s climate is Mediterranean in the north, whereas the south of the country is desert. The country is divided into four regions: the Greater Tunis (24% of the population), the northern region, the central region, and the southern region. Health services are provided by the public and private sectors, with public facilities representing 56% of the total bed capacity in Tunisia, mainly located in “Grand Tunis” (40%) and the Central East (33%) [16].

- Specific setting: AMR surveillance network

AMR surveillance was initiated in 1999 by the “LART” network, “L’Antibio-Résistance en Tunisie,” a group of volunteer microbiologists from university hospitals. During 1999–2010, AMR surveillance was carried out in 4 public sector university hospitals, which expanded to 8 in 2011 and was further extended to 10 in 2012. Since 2019, a total of 11 AMR surveillance sites have been functional: 8 in the Grand Tunis region and 3 in the Central East region.

In 1999, nine bacterial species were being tested for; these were *E. coli*, *K. pneumoniae*, *P. aeruginosa*, *Salmonella* spp., *E. faecalis*, *S. pyogenes*, *S. aureus*, *S. pneumoniae*, and *H. influenzae*. This spectrum has been expanded to include *A. baumannii* and *E. faecalis* in 2008 and *E. cloacae* in 2011. In addition, the capacity of the network for testing and reporting antibiotics has expanded over the years according to international recommendations [12].

### 2.4. Bacterial Species Identification and Antimicrobial Susceptibility Testing

Blood samples are collected from hospitals enrolled in the AMR surveillance system for culture under aseptic conditions from hospitalized patients and outpatients with suspected fever or sepsis. All blood culture vials received at the laboratories are incubated in an automated system (Bact/Alert bioMérieux^®^, Marcy-l’Étoile, France or BD Bactec Blood Culture^®^ Becton, Dickinson and Company, France, depending on the laboratories) for 5 to 7 days.

When a vial is detected as positive by automation, a Gram stain is immediately performed to provide a preliminary idea of the microorganism(s) present. Following this, blood is subcultured on appropriate agar media, including blood agar and chocolate agar (incubated in 5–10% CO_2_), at 35–37 °C for 18–24 h. Bacterial identification is based on morphological characteristics (colony morphology and Gram staining), biochemical tests (manual and automated), and antigenic characteristics: *Enterobacterales*, oxidase-positive Gram-negative bacilli, and *Enterococci* are identified by API (BioMérieux, Marcy-l’Étoile, France) or Vitek^®^2 Compact (BioMérieux, Marcy-l’Étoile, France), staphylococci by tube coagulase; β-haemolytic streptococci by Lancefield antigen testing; and *Salmonella* by serotyping according to the White–Kauffmann–Le Minor scheme by the polyvalent O and H, O4, O9, Hd, Hg, Hi, Hm, and Vi antisera. This process of identifying bacterial species takes approximately 24–48 h.

Depending on the surveillance site, antibiotic susceptibility is determined either through disc diffusion on Mueller Hinton agar or by automated techniques (Vitek^®^2 Compact (BioMérieux, Marcy-l’Étoile, France), according to the guidelines of the “Comité de l’antibiogramme de la Société Française de Microbiologie” (CA-SFM) and the European Committee on Antimicrobial Susceptibility Testing (EUCAST) that were applicable at the time of sample collection [12,17].

Interpretation of susceptibility is performed according to the CA-SFM/EUCAST guidelines, using the version at the time of isolate testing over the 13-year study period.

All the laboratories participating in the surveillance program followed a comparable methodology regarding bacterial species identification, antibiotic susceptibility testing, and interpretation.

### 2.5. Recording and Reporting System

The results of blood culture testing were entered daily into the LIS by a microbiologist in the respective laboratory. Aggregate data for specific indicators were extracted annually from the LIS in Excel format and entered into the surveillance network database at each laboratory. This surveillance database, which receives aggregate data from 11 sentinel surveillance sites in Tunisia, was the source of data for this study.

### 2.6. Data Collection and Variables

Between 2011 and 2023, aggregate data on blood culture isolates of bacterial pathogens and their resistance profiles were downloaded from a surveillance database. To avoid duplication, we only considered the first isolated strain isolated from the patient for the bacteraemia episode.

The following annual aggregate data were extracted: total number of isolates positive for bacterial pathogens, number of isolates positive by the type of bacterial pathogen, number of bacterial isolates tested for resistance to selected antibiotics (ESKAPE pathogen-wise), number of bacterial isolates resistant to selected antibiotics (ESKAPE pathogen-wise), and number of bacterial isolates positive for specific AMR phenotypes.

The list of antibiotic–pathogen combinations is given below:

- *E. cloacae*, *K. pneumoniae*: Amoxicillin-clavulanic acid (AMC), cefotaxime (CTX), gentamicin (GEN), amikacin (AMK), imipenem (IMP), ertapenem (ERT), and ciprofloxacin (CIP).
- *S. aureus*: Cefoxitin (FOX), GEN, AMK, ofloxacin (OFL), vancomycin (VAN), and linezolid (LIN).
- *E. faecium*: Ampicillin (AMP), gentamicin high-charged (GHC), VAN, LIN, and tigecycline (TIG).
- *P. aeruginosa* and *A. baumannii*: Piperacillin–tazobactam (PTZ), ceftazidime (CAZ), GEN, IMP, CIP, and AMK.

### 2.7. Data Analysis

Aggregate data from each hospital in Excel format were collated and imported into STATA v.13 for analysis. Trends in the number of positive bacterial isolates and the proportion of bacterial isolates resistant to selected antibiotics (pathogen-wise) are depicted as line diagrams. The proportion of bacterial isolates resistant to various antibiotics (pathogen-wise) is shown using vertical bar diagrams. The distribution of bacterial pathogens responsible for BSI among all the positive isolates is displayed using a stacked vertical bar diagram. The Cochran–Armitage test with continuity correction was used to test linear trends in the proportion of bacterial isolates (ESKAPE pathogens) resistant to selected antibiotics and specific AMR phenotypes, which yields a *p*-value. A *p*-value < 0.05 represents a statistically significant linear trend.

## 3. Results

### 3.1. Trends in Bacterial Isolates

**Figure 1:**
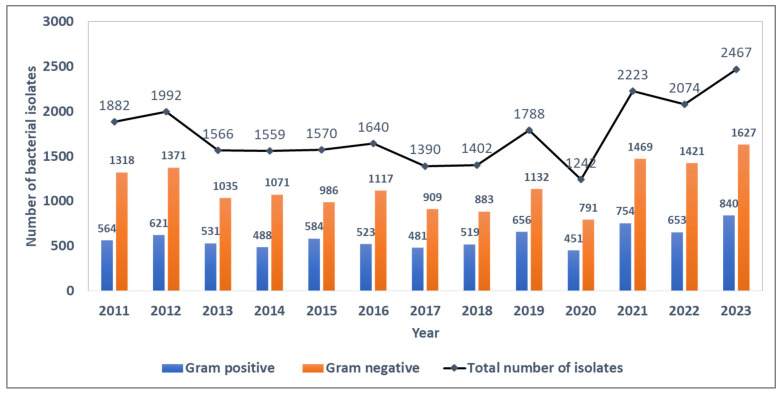
Trends in the number of blood-culture-positive bacterial isolates and the distribution of Gram-positive and Gram-negative isolates from the antimicrobial resistance sentinel surveillance laboratories in Tunisia, 2011–2023.

Between 2011 and 2023, 22,795 blood culture bacterial isolates were identified, of which 7665 (33.6%) were Gram-positive and 15,130 (66.4%) were Gram-negative bacterial strains. The ratio of Gram-negative to Gram-positive strains during the surveillance period was ~2.0, with annual estimates ranging from 1.9 to 2.3 during the surveillance period. The absolute number of bacterial isolates per year was fairly stable until the COVID-19 pandemic in 2020, when the number of isolates showed a sudden dip followed by a sharp rise in subsequent years (Figure 1).

### 3.2. Trends in Distribution of Bacterial Pathogens

**Figure 2:**
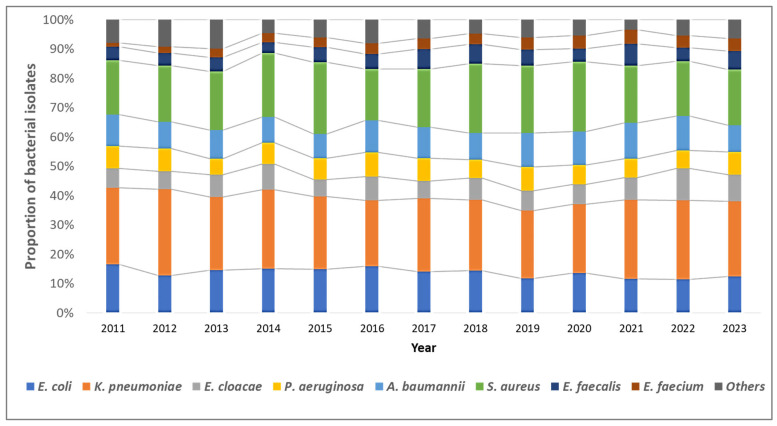
Trends in the distribution of the type of bacterial pathogens among blood-culture-positive bacterial isolates from the antimicrobial resistance sentinel surveillance laboratories in Tunisia, 2011–2023.

Overall, *K. pneumoniae* isolates (*n* = 5830, 38.5%) were the most common, followed by *S. aureus* (*n* = 4650, 20.4%), *E. coli* (*n* = 3098, 13.6%), and *A. baumannii* (*n* = 2352, 10.3%). The distribution of bacterial pathogens isolated from blood cultures remained stable over the study period (Figure 2).

### 3.3. Patterns of Antibiotic Resistance and Their Trends

**Figure 3:**
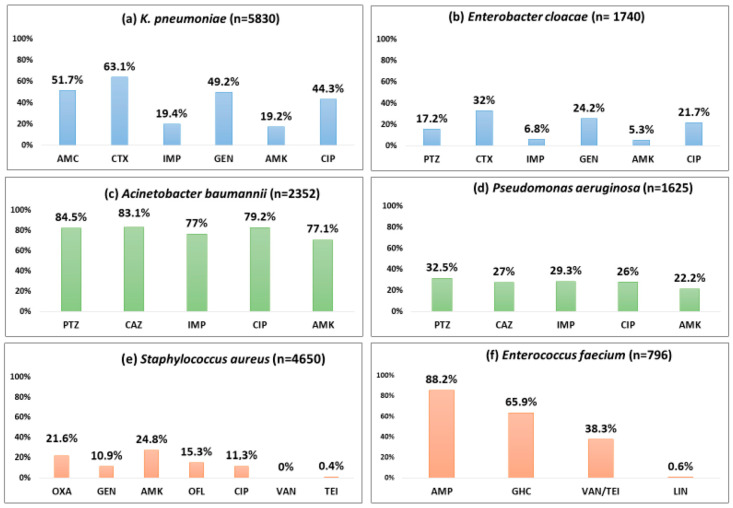
Proportion of blood-culture-positive bacterial isolates resistant to selected antibiotics by the type of pathogen (ESKAPE) from the antimicrobial resistance sentinel surveillance laboratories in Tunisia, 2011–2023. Abbreviation: ampicillin (AMP); amoxicillin-clavulanic acid (AMC); piperacillin–tazobactam (PTZ); cefotaxime (CTX); ceftazidime (CAZ); imipenem (IMP); gentamicin (GEN); gentamicin high-charged 30 µg (GHC); amikacin (AMK); ciprofloxacin (CIP); ofloxacin (OFL); oxacillin (OXA); vancomycin (VAN); teicoplanin (TEI), linezolid (LIN).

**Figure 4:**
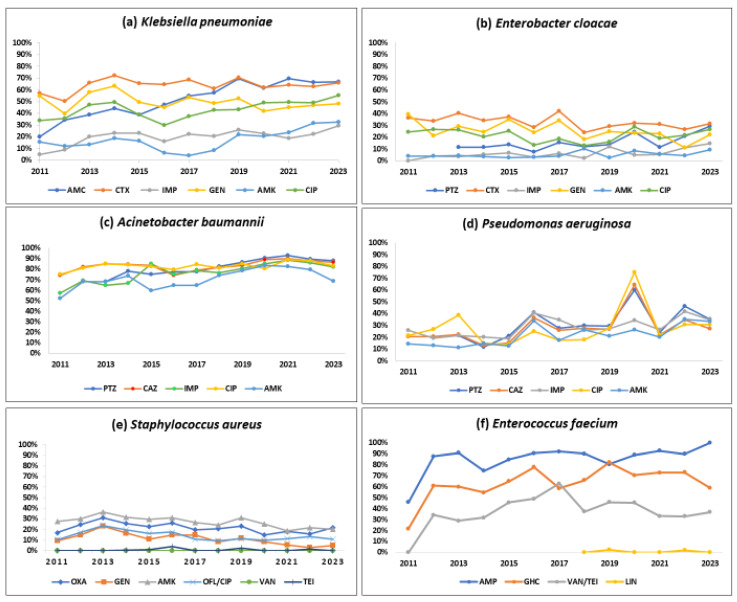
Trends in the proportion of positive bacterial isolates resistant to selected antibiotics by the type of pathogen (ESKAPE) from the antimicrobial resistance sentinel surveillance laboratories in Tunisia, 2011–2023. Abbreviation: amoxicillin-clavulanic acid (AMC); cefotaxime (CTX); imipenem (IMP); gentamicin (GEN); amikacin (AMK); ciprofloxacin (CIP); ofloxacin (OFL); oxacillin (OXA); vancomycin (VAN); teicoplanin (TEI); ampicillin (AMP); gentamicin high-charged 30 µg (GHC); linezolid (LIN).

Table 1 show the number of isolates tested and the number and proportion positive for resistance to various antibiotics by Gram-positive and Gram-negative pathogens. Figure 3 and Figure 4 describe the overall proportion of AMR and the time trends of AMR to selected antimicrobials by the type of ESKAPE pathogens during 2011–2023, respectively.

#### 3.3.1. *Klebsiella pneumoniae*

About half of the *K. pneumoniae* strains reported resistance against amoxicillin-clavulanate (51.7%), gentamicin (49.2%), and ciprofloxacin (44.3%). Cefotaxime and imipenem resistance were seen in 63.1%, and 19.4%, respectively. A statistically significant (*p* < 0.01) rising trend in the resistance rates was observed during the study period for amoxicillin-clavulanate (18.9% to 66.8%), cefotaxime (57.1% to 65.8%), imipenem (4.7% to 29.5%), amikacin (15.7% to 32.3%), and ciprofloxacin (34% to 55.3%).

#### 3.3.2. *Enterobacter cloacae*

One-third of the isolates (32%) were resistant to cefotaxime, a 3GC; 24.2% of these were resistant to gentamicin, and 6.8% were resistant to imipenem. Imipenem resistance was first detected in 2012 in 4% of the *E. cloacae* isolates, and this proportion increased to 14.5% in 2023 (*p*-value for trend < 0.001).

#### 3.3.3. *Acinetobacter baumannii*

More than 80% of the isolates were resistant to piperacillin-tazobactam (84.5%), ceftazidime (83.4%), and ciprofloxacin (79.2%). Resistance to imipenem (also known as CRAB) and amikacin was seen in 77% and 71.1%, respectively with a significant increasing trend over time (*p*-value < 0.01). CRAB rose from 57.2% to 82.1% during 2011–2023 (*p*-value < 0.001).

#### 3.3.4. *Pseudomonas aeruginosa*

Three out of ten isolates were found to be resistant to piperacillin-tazobactam (32.5%), ceftazidime (27%), and imipenem (29.3%), respectively, and one-fifth were resistant to amikacin (22.2%). These rates showed a statistically significant rising trend over the last 13 years (*p* < 0.001). Resistance to piperacillin-tazobactam and amikacin increased from 21.5% to 35.1% and from 14.4% to 33.5%, respectively.

#### 3.3.5. *Stapylococcus aureus*

One out of five *S. aureus* isolates was MRSA (21.6%), and this proportion remained stable over time with no significant trend. *S. aureus* remained susceptible to vancomycin and linezolid with no reported resistance. However, the emergence of resistance to teicoplanin (0.4%) has been reported.

#### 3.3.6. *Enterococcus faecium*

Resistance to ampicillin, vancomycin (also known as VRE), and a high level of resistance to gentamicin were seen in 88.2%, 38.3%, and 65.9% of the isolates, respectively. Resistance to linezolid was reported for the first time in 2019 in 2.4% of the isolates. A statistically significant (*p* < 0.001) rising trend in the resistance rates was observed during the study period for ampicillin (45.8% to 100%), gentamicin (21.7% to 59%), and vancomycin (0% to 36.8%).

#### 3.3.7. Other Specific AMR Phenotypes

CRE and 3GCREB increased from 10.6% to 26.3% (*p*-value < 0.001) and from 39% to 50.2%, respectively, during 2011–2023 (*p*-value < 0.001).

Figure S1 reports AMR pattern to selected antimicrobials among *E.coli* and *E.faecalis* isolates. Figure S2 shows the trends in the proportion of positive *E. coli* and *E. faecalis* isolates resistant to selected antibiotics 2011–2023 (*p*-value < 0.001).

## 4. Discussion

This is the first study from Tunisia comprehensively describing the blood culture bacterial pathogen profile and AMR patterns trends over a 13-year period. There were some interesting findings. First, resistance to carbapenems and 3GC among *A. baumannii* and *Enterobacterales* was high and increased over the last decade, which has important clinical implications, especially for patients in critical settings, such as intensive care units. Second, high vancomycin resistance and the emergence of linezolid resistance in *E. faecium* isolates limit therapeutic options. Third, the stable MRSA resistance rates and vancomycin susceptibility in *S. aureus* isolates are encouraging but necessitate the judicious use of vancomycin for MRSA isolates. Fourth, although the current surveillance system provides useful information on antibiotic resistance trends and patterns, it lacks integration with clinical data to make rapid and meaningful clinical decisions.

CRE increased nearly six times over the last decade in Tunisia. Nearly one out of five *Klebsiella* isolates were imipenem-resistant. This is a significant finding as carbapenems are considered one of the “last-resort” agents in the face of limited treatment options for CRE pathogens. An analysis of surveillance data in the UAE in 2021 reported that carbapenem resistance among *K. pneumoniae* isolates was as high as 67.6%, 76.2%, and 91.6% for imipenem, meropenem, and ertapenem, respectively. CRE has also been associated with higher mortality (relative risk, 6.3) and ICU admission (relative risk, 3.9) [18]. Countries such as Belarus, Georgia, Greece, Moldova, Romania, Russia, Serbia, and Ukraine have also reported CRE in >50% of the isolates [19]. The Global Burden of Disease estimates showed that among Gram-negative bacteria, resistance to carbapenems increased more than any other antibiotic class during 1990–2021 [6].

Along with rising carbapenem resistance, approximately two-thirds of *K. pneumoniae* and one-third of *E. cloacae* isolates are also resistant to cefotaxime (3GC). It is deeply concerning as 3GC drugs are widely used, often with limited access to alternative agents. A systematic review of 28 studies in sub-Saharan African countries found that nearly 54% of *Klebsiella* isolates were resistant to 3GCs [20]. A total of 19 countries in the WHO European Region, particularly in the Southern and Eastern parts of the region, also reported resistance to 3GC in >50% of *Klebsiella* isolates [19]. The high resistance to both carbapenems and 3GCs among *Enterobacterales* is a challenge to patient management and survival, especially among patients with severe infections and those critically ill in ICUs, because they are the cornerstone of antibiotic therapy in such settings.

According to our findings, nearly 80 percent of *Acinetobacter baumannii* strains were found to be resistant to carbapenems (these are known as CRAB), which constitutes a great challenge for clinical practice. Similar rates of resistance to carbapenems (up to 90%) have been reported in other Mediterranean countries, including Tunisia [21,22]. In a recent study in Lebanon, all *A. baumannii* isolates were resistant to carbapenem, with a high 30-day mortality rate of 72% [23]. The present study also highlighted the significant increase in CRAB trends over time, as described worldwide [19,24]. *A. baumannii* has emerged as a significant multidrug-resistant (MDR) pathogen in hospitals, leading to increased length of intensive care unit (ICU) stays and high mortality, which is why the WHO has put the pathogen in the critical group of bacterial priority pathogens [15], for which new antibiotics and vaccines are urgently needed. The long-term persistence of the organism in the hospital environment makes cross-contamination through air and inanimate objects very likely. This emphasizes the importance of environmental cleaning and disinfection, isolation, and enhanced contact precautions to prevent cross-transmission of CRAB.

Regarding *S. aureus*, the study findings are in alignment with the situation in the majority of European Union countries where MRSA seems to be stabilizing or even decreasing [25]. This could be due to the cyclical success of some methicillin-sensitive *S. aureus* clones that tend to replace dominant MRSA clones [26]. The susceptibility of *S. aureus* to vancomycin was encouraging. However, judicious use of this drug for culture-proven MRSA infections that are not susceptible to routine or alternative agents is necessary to prevent the development of vancomycin-resistant *S. aureus.*

More than one-third of the *E. faecium* isolates were vancomycin-resistant (VRE), which is much higher than reported in other countries of the region, such as Egypt and the UAE, with 23.1% and 8.1%, respectively [27,28]. This could be due to the emergence of novel clones of the bacteria in Tunisia, which is a precursor to the rapid evolution of these resistant bacteria [18]. However, a number of other European countries have reported high rates of vancomycin resistance among *E. faecium* isolates, ranging from 27 to 66% [29]. Our study also reported the emergence of linezolid-resistant *E. faecium* isolates as high as 2.4%. With linezolid among the few remaining therapeutic options, this is a major challenge to managing nosocomial infections due to VRE [30,31].

### Strengths and Limitations

The major strength of this study is the analysis of a large number of bacterial isolates (*n* = 22,795) from the nationwide AMR surveillance network. The network covered the majority of the high-burden public sector tertiary teaching hospitals in the country that followed standardized laboratory protocols for testing blood specimens for bacterial identification and antimicrobial testing over a long 13-year period, making the findings representative of what is happening in the country’s public health sector.

However, the study had a few limitations. First, the surveillance network does not capture the scenario in the private sector, which caters to a substantial proportion of the population. Second, we could not calculate and compare incidence rates of BSIs due to a lack of a defined catchment area/population. Third, the lack of individual-level clinico-demographic data, such as age, gender, length of stay, clinical profile, resistance profile, diagnosis, etc., did not allow us to explore the factors associated with AMR and multidrug resistance. The lack of patient outcome data also limits our understanding of mortality associated with AMR. Fourth, using aggregate data for analysis rules out the possibility of data validation.

There are four important implications for policy and practice from this study. First, this nationwide AMR surveillance data over a long period will inform protocols for empirical antibiotic use in common bloodstream bacterial pathogens in Tunisia. Second, treatment practices should be individualized based on susceptibility testing to ensure the most effective antibiotics are used. This requires rapid point-of-care diagnostics for bacterial identification and susceptibility testing to aid quick decision-making by clinicians. Third, infection prevention and control (IPC) measures should be strengthened in healthcare settings to reduce the spread of resistant bacteria. Fourth, the lack of clinical data from patients with drug-resistant infections is a major knowledge gap. The AMR surveillance system in Tunisia must integrate antibiotic resistance data with clinical data through real-time data-sharing platforms that allow for the immediate exchange of information across healthcare providers and settings. This will generate big data and will allow us to use machine learning tools to analyze patterns and trends and make useful predictive models for quick and real-time clinical decision-making, and this will eventually lead to improved patient outcomes. User-friendly dashboards that visualize integrated data for clinicians, microbiologists, and public health officials can be helpful.

## 5. Conclusions

The increasing resistance to critically important antibiotics, particularly carbapenems and third-generation cephalosporins (3GCs) among Gram-negative bacteria responsible for bloodstream infections (BSIs), presents a significant and urgent challenge to the management of these infections in Tunisia, as it severely restricts effective therapeutic options. Furthermore, the emerging resistance to linezolid among Gram-positive cocci constitutes an additional substantial threat, necessitating urgent interventions to limit the irrational use of these life-saving drugs. Despite certain limitations, including the scope of the surveillance network and the lack of patient-level clinical information, Tunisia’s national AMR surveillance network has supplied essential foundational data for tracking annual AMR trends and informing empirical antibiotic prescriptions, rational antibiotic use, and stewardship policy in the country.

## Figures and Tables

**Table 1:** Number of ESKAPE blood culture Gram-negative isolates tested positive for resistance to selected antibiotics by the type of pathogen from the antimicrobial resistance surveillance laboratories in Tunisia during 2011–2023.

(**a**)					
**Antibiotics**		***K. pneumoniae*** **(*n* = 5830)**	***E. cloacae*** **(*n* = 1740)**	***A. baumannii*** **(*n* = 2352)**	***P. aeruginosa*** **(*n* = 1625)**
AMC	T	5170	NA	NA	NA
	R	2675	NA	NA	NA
	%	51.7	NA	NA	NA
PTZ	T	-	1235	1521	1229
	R	-	212	1286	399
	%	-	17.2	84.5	32.5
CTX	T	5207	1638	NA	NA
	R	3284	524	NA	NA
	%	63.1	32	NA	NA
CAZ	T	5207	-	2023	1535
	R	3191	-	1688	414
	%	61.3	-	83.4	27
IMP	T	5089	1638	2145	1564
	R	990	111	1651	458
	%	19.4	6.8	77	29.3
ERT	T	5117	1625	NA	NA
	R	1394	197	NA	NA
	%	27.2	36.7	NA	NA
GEN	T	4777	1488	2350	1405
	R	2353	360	1799	393
	%	49.2	24.2	76.5	28
AMK	T	5427	1631	2080	1529
	R	1041	86	1480	341
	%	19.2	5.3	71.1	22.2
CIP	T	4966	1615	2010	1533
	R	2199	350	1671	397
	%	44.3	21.7	79.2	26
(**b**)					
**Antibiotics**		***S. aureus*** **(*n* = 4650)**			***E. faecium*** **(*n* = 796)**
OXA	T	4515			NA
	R	975			NA
	%	21.6			NA
AMP	T	-			761
	R	-			671
	%	-			88.2
GEN	T	4056			719
	R	444			474
	%	10.9			65.9 *
AMK	T	3967			-
	R	986			-
	%	24.8			-
OFL **	T	2875			-
	R	441			-
	%	15.3			-
CIP	T	1175			-
	R	133			-
	%	11.3			-
VAN	T	2011			768
	R	0			294
	%	0			38.3
TEI	T	2013			768
	R	8			294
	%	0.4			38.3
LIN	T	3770			353
	R	0			2
	%	0			0.6

NA: Not applicable; (-): not reported. Abbreviation: AMC = Amoxicillin-clavulanicacid, PTZ = Piperacillin-tazobactam, CTX = Cefotaxim, CAZ = Ceftazidim, IMP = Imipenem, ERT = Ertapenem, GEN = Gentamicin, AMK = amikacin, OXA = oxacillin, AMP = ampicillin, OFL = ofloxacin, CIP = ciprofloxacin, LIN = linezolid, VAN = vancomycin, TEI = teicoplanin. * High level of resistance to gentamicin. ** Ofloxacin was tested and reported for *Staphylococci* until 2019. In 2020, ciprofloxacin was tested and reported instead of ofloxacin. T: tested, number of positive blood cultures isolates tested for each antibiotic. R: resistant, number (*n*) of isolates tested, and proportion (%) of isolates with resistance to each antibiotic relative to the total number of isolates tested for each pathogen each year.

## Data Availability

The data presented in this study are available on request from the corresponding author.

## Supplementary Materials

The following supporting information can be downloaded at https://www.mdpi.com/article/10.3390/tropicalmed10060164/s1. Figure S1 reports the AMR pattern to selected antimicrobials among *E. coli* and *E. faecalis* isolates. Figure S2 shows the trends in the proportion of positive *E. coli* and *E. faecalis* isolates resistant to selected antibiotics 2011–2023 (*p*-value < 0.001).

## Abbreviations

The following abbreviations are used in this manuscript:

AMR	Antimicrobial resistance
AMC	Amoxicillin-clavulanic acid
AMK	Amikacin
AMP	Ampicillin
BSI	Bloodstream infections
CIP	Ciprofloxacin
CRE	Carbapenem-resistant *Enterobacterales*
CRAB	Carbapenem-resistant *A. baumannii*
CTX	Cefotaxime
CAZ	Ceftazidime
ERT	Ertapenem
DALYs	Disability-Adjusted Life Years
ESKAPE	*E. cloacae*, *S. aureus*, *K. pneumoniae*, *A. baumannii*, *P. aeruginosa*, *E. faecium*
FOX	Cefoxitin
GEN	Gentamicin
GHC	Gentamicin high-charged
GLASS	Global Antimicrobial Resistance and Use Surveillance System
ICU	Intensive care unit
IMP	Imipenem
LART	L’Antibiorésistance en Tunisie
LIN	Linezolid
LIS	Laboratory information system
MDR	Multidrug resistant
MENA	Middle East and North Africa
MRSA	Methicillin-resistant *S. aureus*
OFL	Ofloxacin
PTZ	Piperacillin–tazobactam
TIG	Tigecycline
3GC	Third-generation cephalosporin
3GCREB	Third-generation cephalosporin-resistant *Enterobacterales*
VAN	Vancomycin
VRE	Vancomycin-resistant *Enterococci*
WHO	World Health Organization
