# Supplementary material for: Bacterial Pathogens and Antibiotic Resistance in Bloodstream Infections in Tunisia: A 13-Year Trend Analysis
Source: Trop Med Infect Dis. 2025 Jun 12;10(6):164. doi: 10.3390/tropicalmed10060164 (PMC12197613; doi:10.3390/tropicalmed10060164)
Supplement: Supplementary file 1 [file tropicalmed-10-00164-s001.zip › tropicalmed-3612854-supplementary.pdf]

Supplementary appendix

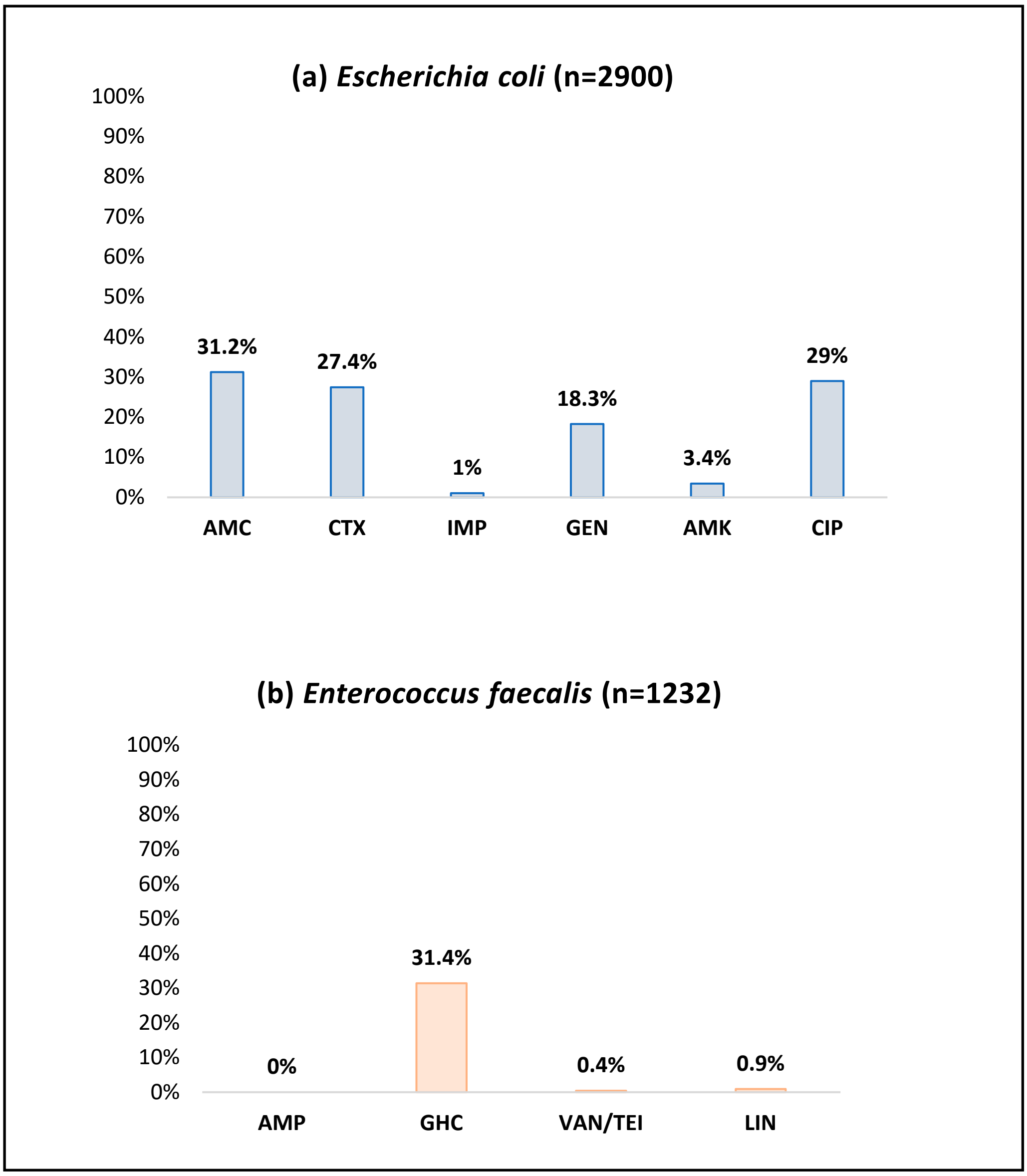

Figure S1. Proportion of blood culture positive *E. coli* and *E. faecalis* isolates resistant to selected antibiotics from the AntiMicrobial Resistance sentinel surveillance laboratories in Tunisia, 2011–2023. Abbreviation: Ampicillin (AMP), Amoxicillin-Clavulanic acid (AMC); Cefotaxime (CTX); Imipenem (IMP); Gentamicin (GEN); Gentamicin high charged 30µg (GHC), Amikacin (AMK); Ciprofloxacin (CIP); Vancomycin (VAN); Teicoplanin (TEI); Linezolid (LIN)

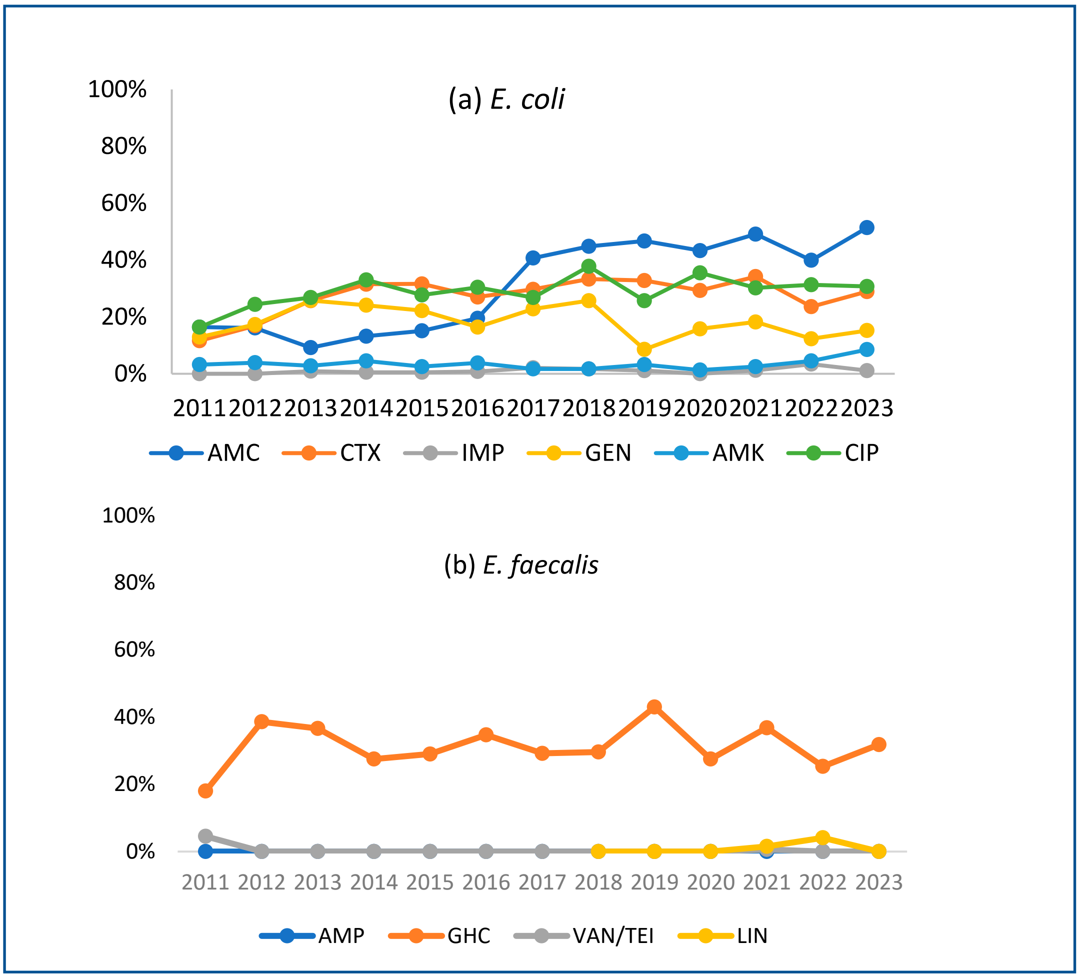

Figure S2. Trends in the proportion of positive E. coli and E. faecalis isolates resistant to selected antibiotics from the Antimicrobial Resistance sentinel surveillance laboratories in Tunisia, 2011–2023. Abbreviation: Ampicillin (AMP), Amoxicillin-Clavulanic acid (AMC); Cefotaxime (CTX); Imipenem (IMP); Gentamicin (GEN); Gentamicin high charged 30µg (GHC), Amikacin (AMK); Ciprofloxacin (CIP); Vancomycin (VAN); Teicoplanin (TEI); Linezolid (LIN)
